# Welcome to *Health Information Science and Systems*

**DOI:** 10.1186/2047-2501-1-1

**Published:** 2013-01-10

**Authors:** Yanchun Zhang

**Affiliations:** Centre for Applied Informatics, College of Engineering and Science, Victoria University, Melbourne, Australia

## Abstract

*Health Information Science and Systems* is an exciting, new, multidisciplinary journal that aims to use technologies in computer science to assist in disease diagnoses, treatment, prediction and monitoring through the modeling, design, development, visualization, integration and management of health related information. These computer-science technologies include such as information systems, web technologies, data mining, image processing, user interaction and interface, sensors and wireless networking and are applicable to a wide range of health related information including medical data, biomedical data, bioinformatics data, public health data.

## Editorial

It is my pleasure to announce the launch of *Health Information Science and Systems* (*HISS*). *HISS* is an exciting, multidisciplinary journal that aims to use technologies in computer science to assist in disease diagnoses, treatment, prediction and monitoring through the modeling, design, development, visualization, integration and management of health related information. These computer-science technologies include such as information systems, web technologies, data mining, image processing, user interaction and interface, sensors and wireless networking and are applicable to a wide range of health related information including medical data, biomedical data, bioinformatics data, public health data and so forth. We hope that *HISS*, as an open access journal, will attract more readers, provide a useful contribution to the scientific literature with its associated citations [1, 2], and help authors in sharing their latest development to a widest possible audience in the fastest and most effective way.

Health and medicine research covers a broad spectrum of research topics, including traditional medical and newly emerging research areas. Particularly, some new interdisciplinary research topics, such as health informatics or e-health, the intersectional domain of Information and communications technology (ICT) and health, has shown great potential in drawing new knowledge and evidence. In the last decades, with the development of database systems and networking technologies, Hospital Information Management Systems (HIMS) and web based clinical or medical systems (such as the Medical Director, a generic GP clinical system) have been widely used in health and clinical practices. In addition, health care and medical service is becoming more data-intensive and evidence-based due to electronic health records being used to track individuals’ and communities’ health information (particularly changes in health information). These highlights substantially motivate and advance the emergence and the progress of health informatics research and practice. The ‘Health Informatics’ discipline is gaining increasing interests from not only academia but also health industries due to the significant initiatives of using information, knowledge and communication technologies in health industries to assure patient safety, improve population health and facilitate the delivery of healthcare services by governments. As a journal, *HISS* is intended to meet the need for cross disciplinary research in IT and health/medical science.

The first articles published in *HISS* comprise four papers (in addition to this Editorial) and the authors are from Singapore, Hungary, Australia, USA and China. These initial articles illustrate how bioinformatics is applied in healthcare and medical management, and highlight some of the emerging research in analysis, sharing and delivering of medical images.

“How bioinformatics influences health informatics: Usage of bimolecular sequences, expression profiles and automated microscopic image analyses for clinical needs and public health”, by Vladimir Kuznetsov, Hwee Kuan Lee, Sebastian Maurer-Stroh, Maria Judit Molnar, Sandor Pongor, Birgit Eisenhaber and Frank Eisenhaber [3], presents the state-of-the-art techniques that may address the problem of translating the human genomic information into phenotypes for personalized treatment through life science research, genomic sequencing and information technology. Particularly, their discussions focus on three aspects: (1) using biomarkers for cancer classification, early diagnostics, prognosis and personalized therapy; (2) analyzing biomolecular sequences and structures for pathogenic viruses and bacteria and their role in combating infections through information technology, such as comparative genomics, 3D structural modeling, literature text mining and plotting geo-temporal occurrence patterns for epidemiological significance; and (3) developing computational methods to analyze bioimages of dermatology, eye diseases and 3D tumor segmentation for assisted surgery. They also point out that the link between biomolecular and clinical data of populations with geographic information can benefit public health.

“A patient-centric distribution architecture for medical image sharing”, by Liviu Constantinescu, Jinman Kim, Ashnil Kumar, Daiki Haraguchi, Lingfeng Wen and Dagan Feng [4], presents a distribution architecture for medical image display across numerous devices and media. In this architecture, a preprocessor and an in-built networking framework are used to improve compatibility and promote greater accessibility of medical data. They also develop an INVOLVE2 system, comprising a preprocessor for collating and converting imaging studies into a compressed and distributable format, a workflow for self-managing distribution of medical data via CD, USB and network, and an interface for potential mobile and web-based data access. The advantages of INVOLVE2 are: (1) outpatient users can comfortably access and interpret their own data, and (2) a friendly user-interface for image viewing by both outpatients and referring physicians, provides a digital image access via mobile devices or web.

“iPathCase^KEGG^: An ipad interface for KEGG metabolic pathways”, by Stephen R. Johnson, Xinjian Qi, A Ercument Cicek and Gultekin Ozsoyoglu [5], provides an iPad interface - iPathCase^KEGG^ for Kyoto Encyclopedia of Genes and Genomes (KEGG) data. KEGG links sequenced genomes to higher level systematic functions but has a very limited web access interface. With a tablet interface that have multiple browsing and visualization capabilities to support mobile access to KEGG, iPathCase^KEGG^ allows users to pick pathways, visualize them and see detail pages of reactions and molecules using the multi-touch interface of iPad and to interact with the data in multiple ways. iPathCase^KEGG^ is a convenient tool for life science instructors and researchers.

“Segmentation of ultrasound images of thyroid nodule for assisting fine needle aspiration cytology”, by Jie Zhao, Wei Zheng, Li Zhang and Hua Tian [6], describes an efficient and accurate method of segmenting thyroid nodule from ultrasound images. In this method, they combine the anisotropic diffusion model with the normalized cut to improve the image quality by removing the speckle noise produced in the formation process of B ultrasound image of thyroid tumor. Since they only preserve the important edges and local details of the B ultrasound image without noises through the enhancement of anisotropic diffusion model, both the efficiency of the computation for constructing the weight matrix of the improved normalized cut and the accuracy of the final segmentation results are increased.

Currently, the editorial board comprises 28 leading scientists and researchers including 24 associate editors [7]. Manuscripts will be assigned to a handling editor who will oversee the review process and make recommendations on acceptance to the Editor-in-Chief, who will then make the final editorial decision. For manuscripts deemed suitable for peer review two or more expert reviews will be sought. It takes an average of 8 weeks from submission to the first decision and 3-4 weeks to final decision after authors submitting their revised versions. Manuscripts are published immediately the production process has been completed, with their final citation, and no need to wait for assignment to an issue. Published articles are submitted to a number of databases and indexing services, with more that can be added now that we have launched.

I congratulate the Managing Editors, Deputy Editors and Associate Editors and reviewers of *HISS* for a job well done. I also thank the authors for sending high quality manuscripts for publication to our journal. I wish everyone a happy and flourishing new year.

I also invite comments and suggestions from authors, reviewers and readers on how to improve the quality of *HISS*. I also seek your help in spreading the word about *HISS* to your colleagues. I look forward to hearing from you.

Yanchun Zhang

Editor-in-Chief
